# Optimization of a simple, accurate and low cost method for starch quantification in green microalgae

**DOI:** 10.1186/s40529-019-0273-y

**Published:** 2019-10-13

**Authors:** Tze Ching Yong, Chia-Sheng Chiu, Ching-Nen Nathan Chen

**Affiliations:** Department of Oceanography, National Sun Yat-sen University, Kaohsiung, 804 Taiwan

**Keywords:** Microalgae, Photosynthate partitioning, Starch quantification, Biofuels

## Abstract

**Background:**

Lipids and starch are important feedstocks for bioenergy production. Genetic studies on the biosyntheses of lipids and starch in green microalgae have drawn significant attention recently. In these studies, quantifications of lipids and starch are required to clarify the causal effects. While lipids are assayed with similar procedures worldwide, starch in green microalgae has been measured using various methods with deficiencies in accuracy or high cost.

**Results:**

A simple, accurate and low cost procedure for routine quantification of starch in green microalgae was developed. This procedure consists of quick-freezing of the cells, solvent extraction of the pigments, 134 °C autoclaving and glucoamylase double digestions of starch, followed by a glucose assay using the dinitrosalicylic acid reagent. This procedure was optimized to quantify starch in small volumes of green microalgal culture. The accuracy of starch quantification using this procedure was 102.3 ± 2.5% (mean ± SD, n = 6), as indicated by using cornstarch as internal controls. The working protocol is available at http://dx.doi.org/10.17504/protocols.io.2mhgc36.

**Conclusions:**

This quantification approach overcomes the current problems in the starch quantification of green microalgae such as inaccuracy and high cost. This approach would provide an opportunity to compare the effects of genetic, physiological or cultivation manipulations on the productivity of starch in green microalgae elucidated in different labs, which is essential in the enhancement of lipid productivity studies in microalgae.

## Background

Biodiesel is superior to bioethanol in terms of production considerations, and their raw materials are lipids and starch, respectively (Chisti 2008). Although some oleaginous green microalgae accumulate high levels of lipids in their cells, a significant drawback found in them is that their energy reservoir includes starch that is less desirable as a feedstock for biofuel production. In the production of bioethanol, the raw materials containing starch have to be hydrolyzed into glucose first, followed by anaerobic fermentation, centrifugation and distillation to produce and concentrate bioethanol. In this procedure, one-third of the glucose carbon is lost and it takes a significant amount of energy input. On the other hand, to produce biodiesel, the storage lipid triacylglycerol simply goes through transesterification and the products are glycerol and biodiesel.

It has been speculated that starch biosynthesis must be suppressed in order to enhance lipid productivity in green microalgae (Siaut et al. 2011). The rationale behind this thought is that biosyntheses of the two kinds of molecules compete for the same precursor 3-phosphoglycerate (3-PG). Genetic modification approaches have been taken to change the metabolite flux in green microalgae recently. To verify whether these approaches work to reduce starch biosynthesis at the same time enhancing lipid productivity in green microalgae, a simple, accurate and low cost method for starch quantification is required for the routine measurements. While methods for lipid extraction and measurement have reached a consensus worldwide (based on organic solvent extraction followed by transesterification and GC analysis) (Bligh and Dyer 1959; Folch et al. 1957; Pan et al. 2011), starch measurement is still practiced in various ways in different labs to date. In 1991, Rose et al. compared six starch quantification methods that employed either perchloric acid extraction or starch-digesting enzymes. Their results demonstrated that the variations of these methods could reach 20 to 40% (Rose et al. 1991). Steps described in those methods are still adopted for microalgal starch assay nowadays. In the recent microalgal literature, methods for starch quantification include acid hydrolysis of starch followed by color formation using anthrone or HPLC analysis of glucose (Branyikova et al. 2011; Kato et al. 2017), amylase/amyloglucosidase digestion of starch followed by glucose oxidase reaction and spectrophotometry (Dragone et al. 2011), and assay kits from Sigma-Aldrich, USA (Cat. # SA20; USD 153 for 20 assays sold in the US; USD 284 in Taiwan) and Megazyme, Ireland (Cat. # K-TSHK, Euro 263 for 100 assays), respectively (Juergens et al. 2016; Soh et al. 2014). These different methods result in different accuracies for starch quantification. Starch quantification using acid hydrolysis at a high temperature could lead to over-estimate of the actual starch level in the cells because this reaction could also hydrolyze other glucose-containing polysaccharides and glycoproteins in the cells. The approach using amylase/amyloglucosidase digestion followed by glucose oxidase actually uses the third enzyme peroxidase and the chemical *o*-dianisidine to produce color for the spectrophotometric measurement. The three enzyme reactions compromise the simplicity and accuracy of this approach in addition to the cost considerations. The most costly methods involve the use of commercial assay kits, which are unlikely to be adopted for routine assays.

To overcome these barriers, a simple, accurate and low cost procedure was developed for quantification of starch in green microalgae. In this procedure, a thermo-tolerant enzyme glucoamylase (EC 3.2.1.3, Tokyo Chemical Industry, more information available in BRENDA database) and the chemical dinitrosalicylic acid were adopted (Miller 1959; Saqib and Whitney 2011; Wang et al. 1997). This procedure requires a small amount of cell culture only, which well fits lab-scale microalgal cultivation. The procedure and the verification of this procedure’s accuracy are presented here.

## Materials and methods

### Working protocol webpage in protocols.io

The working protocol is available at http://dx.doi.org/10.17504/protocols.io.2mhgc36.

### Microalga and cultivation

*Chlamydomonas reinhardtii* UTEX 90, a wildtype strain, was purchased from the Culture Collection of Algae at the University of Texas at Austin, USA. The cells were propagated under continuous 150 μmol photon/m^2^/s white light at 25 °C in a modified Bold 3 N medium which contains 1.1 mM NaNO_3_, 0.05 mM K_2_HPO_4_, 0.16 mM KH_2_PO_4_, 0.17 mM CaCl_2_, 0.3 mM MgSO_4_, 0.43 mM NaCl and minerals including 6.56 µM FeCl_3_, 0.25 µM ZnSO_4_, 2.42 µM MnSO_4_, 5.69 nM CoSO_4_, 6.1 nM Na_2_MoO_4_, 1 nM Na_2_SeO_3_, 6.3 nM NiCl_2_, described in Table 2 of Berges et al. (2001).

### Chemicals and enzyme

Corn starch (S5296), glucose (G5146), dinitrosalicylic acid (D0550), NaOH (S8045), and NaH_2_PO_4_ (S0751) were purchased from Sigma-Aldrich, USA. Glucoamylase (*a.k.a.* amyloglucosidase, EC 3.2.1.3) was purchased from Tokyo Chemical Industry, Japan (Cat. # M0035, from *Rhizopus* sp., about 6000 units/g, 25 g sold for USD 165 in Taiwan). This enzyme completely hydrolyzes soluble starch, amylose, and amylopectin (see in BRENDA database). Potassium sodium tartrate (131,729.1210) was purchased from PanReac AppliChem, Spain.

### Glucose assay and calculation

**Fig. 1:**
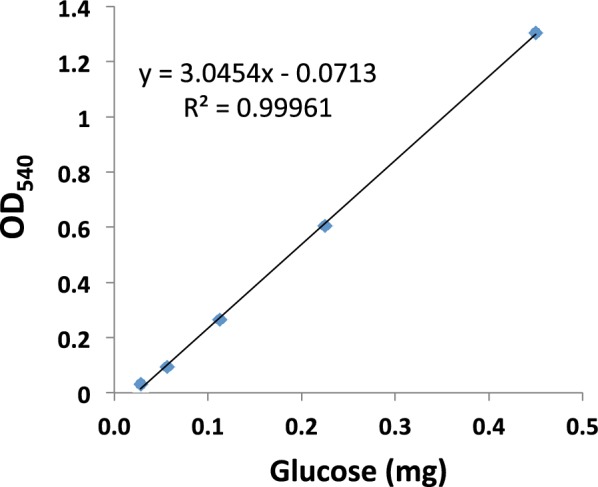
A standard curve of the glucose assay generated using the dinitrosalicylic acid method

Glucose was dehumidified and weighed using a high accuracy analytical balance (METTLER AT21, Columbus, OH, USA; readability to 5 μg). A solution of 10 mM was prepared and stored at 4 °C. Serially twofold diluted glucose solutions, 0.5 mL each, were mixed with 2 mL DNS reagent (44 mM dinitrosalicylic acid, 1 M potassium sodium tartrate, and 0.5 M NaOH) separately and then heated in boiling water for 5 min. After cooling in tap water, the optical density at 540 nm (OD_540_) of each mix was measured. A standard curve was built based on the glucose quantity in each mix against its OD_540_ (Fig. 1). Samples of 0.5 mL were treated and measured using the same procedure in parallel to the standards in each batch of measurement. The glucose quantity in each sample was calculated based on the regression equation of the standard curve. The level of experimental replications was indicated in each Table. The results were analyzed using *t* test with 95% confidence interval.

### Complete disintegration of starch granules, enzymatic digestion and calculation

Each corn starch sample, weighed using the high accuracy analytical balance aforementioned and the quantities specified in Table 1, was mixed with 5 mL sodium phosphate buffer (50 mM, pH 5.0). The samples were disintegrated using autoclaving at 134 °C for 1 h. Glucoamylase powder was dissolved in the same buffer to make 100 units per mL. Two units of the glucoamylase (in 20 μL) were mixed with 0.5 mL of the autoclaved starch solution plus 0.5 mL of the phosphate buffer, and the enzymatic digestion was executed at 50 °C overnight. A second digestion was executed in the same conditions for 7 h by adding the same amount of enzyme to the mix. In the course of the method setting (the pilot tests), each of the reactions was mixed with 20 μL KI-I_2_ reagent in a time-course serial to inspect the remaining starch using spectrophotometry at OD_590_. The enzymatic reaction is shown below.

$$ \begin{aligned}   \left( {n{\text{Glucose~in~polymer}}} \right)~ +  & \, \left( {H_{2} O} \right) n - 1~\xrightarrow{{{\text{Glucoamylase}}~{\text{at}}~50\;^{ \circ } {\text{C}}}}\left( {{\text{Glucose}}} \right)  n \\     & M.W. = 18~\quad \quad \quad \quad \quad \quad \quad \quad M.W. = 180 \\  \end{aligned}  $$

**Table 1:** Effects of autoclaving temperature and enzymatic digestion duration on the accuracy of starch measurement using this method

	Starch raw weight (μg)	Starch net weight (μg)^e^	Starch quantity measured by this method (μg)	Accuracy (%)	Mean ± SD
121 °C autoclave and single digestion	15,458	13,186	12,037	91.3	
	15,555	13,268	12,266	92.5	
	15,227	12,989	11,769	90.6	
	15,237	12,997	11,479	88.3	90.7 ± 1.7^a^ (n = 4)
121 °C autoclave andouble digestions	20,462	17,454	16,762	96.0	
	24,678	21,050	19,542	92.8	
	29,582	25,233	23,455	93.0	
	32,898	28,062	25,863	92.2	93.5 ± 1.7^a,b^ (n = 4)
134 °C autoclave and single digestion^f^	20,593	17,566	16,133	91.9	
	21,107	18,004	17,596	97.7	
	26,987	23,020	21,999	95.6	
	30,476	25,996	24,779	95.3	
	35,679	30,434	28,459	93.5	94.8 ± 2.2^b,c^ (n = 5)
134 °C autoclave and double digestions^f^	20,593	17,556	17,282	98.4	
	21,107	18,004	17,760	98.6	
	26,987	23,020	23,089	100.3	
	30,476	25,996	25,461	97.9	
	35,679	30,434	30,149	99.1	98.9 ± 0.9^d^ (n = 5)

The letters a, b, c and d after mean ± SD indicate significant difference (*p* < 0.05, *t*-test)

^e^The net weight excluded the weight of the water, protein and fiber in the corn starch

^f^The samples after autoclaving were split for single digestion and double digestions

The glucose product was measured using the dinitrosalicylic acid (DNS) method described in the previous “Glucose assay and calculation” section. The net weight of the corn starch (glucose in polymer) was determined by multiplying the quantity of the glucose product by 0.9.

### Microalgal sample preparation—pigment extraction by using methanol-tetrahydrofuran

Ten microliter of the day 5 microalgal culture (OD_682_ around 1.0) were harvested using swing bucket centrifugation (2600*g*, 3 min) at room temperature. The supernatant was discarded and the cells were transferred to a 2-mL screw cap microtube rapidly. After a brief high-speed centrifugation, the supernatant in the microtube was removed using a pipette and the cells were quickly frozen at − 15 °C in a mix of ice and crude sea salt. One microliter of cold methanol/tetrahydrofuran (v/v = 1/3) was added to the frozen cells. The cells were soaked in the solvent for 30 min in the ice-salt mix and agitated occasionally to extract the pigments. After centrifuging at 16,000*g* at 4 °C for 10 min, the solvent was discarded and the pellet was dried at 65 °C for 1 h. The dried pellet was repeatedly washed out using the sodium phosphate buffer (50 mM, pH 5.0) and the final volume was adjusted to 5 mL. A small amount of corn starch, weighed using the high accuracy analytical balance aforementioned and listed in Table 2, was added to each suspension serving as the internal control. This mix was autoclaved at 134 °C for 1 h. One mL of the autoclaved sample was smashed using a mini-beadbeater (BioSpec Products Inc., USA) to fully release the starch from the cells, and 0.5 mL of the smashed sample was mixed with 0.5 mL of the sodium phosphate buffer then subjected to the double digestions aforementioned. After the double digestions, the sample was centrifuged again to precipitate any cell debris. Five hundred microliter of the supernatant was used for glucose measurement as described in the previous “Glucose assay and calculation” section.

**Table 2:** Accuracy of measuring the internal control cornstarch mixed with algal samples using this method

Net weight of internal control cornstarch (μg)^a^	Algal starch quantified using this method (μg)	Combined starch in the mix of algal cells and internal control quantified using this method (μg)	Internal control determined using this method (μg)^b^	Accuracy of internal control measurement (%)^c^
0	4993			
1240		6290	6290–4993 = 1297	104.6
0	4993			
3917		9018	9018–4993 = 4025	102.8
0	4993			
5522		10,802	10,802–4993 = 5809	105.2
0	5804			
1051		6836	6836–5804 = 1032	98.2
0	5804			
4033		9917	9917–5804 = 4113	102.0
0	5804			
5748		11,613	11,613–5804 = 5809	101.1

^a^The net weight excluded the weight of the water, protein and fiber in the cornstarch

^b^The quantities were obtained by subtracting the amount of algal starch measured in the controls (without the cornstarch internal control) from the total starch

^c^Mean ± SD is 102.3 ± 2.5% (n = 6)

## Results

### Estimate of the net weight of glucose polymers in the corn starch

As in the grains of crops, corn starch contains certain levels of water, protein and cellulosic fiber that affect the measurement of the net weight of starch (glucose polymer). Corn starch samples, stored in the lab refrigerator, weighing more than 0.3 g were dried at 105 °C for 2 h in a ceramic crucible. The weight difference of each sample before and after the drying was measured using a high accuracy analytical balance. The water content in the starch was determined to be 13.5 ± 1.3% (mean ± SD, n = 3). The contents of protein and fiber in the corn starch were measured by the U.S. Department of Agriculture (https://ndb.nal.usda.gov/ndb/foods/show/305228), which together comprised 1.16% raw weight of corn starch. The amounts of other components such as lipids and minerals were very little as shown in our results and in the USDA database. They were thus ignored in the net weight calculation. Therefore, the net weight of starch (glucose polymer) was 85.3% of the raw weight of the corn starch used in this study.

### Effects of autoclaving temperature and duration of enzymatic digestion on starch degradation

Two parameters, autoclaving temperature and duration of enzymatic digestion, can affect the completeness of starch digestion by glucoamylase. Starch granules are formed by compacted glucose polymers in the cells of green algae and plants. The compact structure hinders enzymatic reactions and thus it has to be disintegrated before the enzyme can completely digest the glucose polymers. The effect of autoclaving temperature on the disintegration of starch granules was examined. In addition, the duration of the enzymatic digestion of dissolved glucose polymers is an important factor that determines the accuracy of starch measurement. This factor was also investigated in this study.

As shown in Table 1, the highest accuracy of the starch measurement using this procedure was achieved by autoclaving at 134 °C in conjunction with the glucoamylase double digestions (98.9 ± 0.9%, n = 5), followed by autoclaving at 134 °C coupled with single digestion (94.8 ± 2.2%, n = 5) and autoclaving at 121 °C with double digestions (93.5 ± 1.7%, n = 4. It should be noted that the difference between the two sets of measurements was not statistically significant), and lastly autoclaving at 121 °C with single digestion (90.7 ± 1.7%, n = 4).

### Estimate of the accuracy of measuring endogenous starch in algal cells

Unlike processed corn starch, microalgal cells contain pigments that could impede the starch measurement which is based on spectrophotometry at 540 nm absorption. Pigment extraction seems to be the best option to avoid this problem. The solvent methanol/tetrahydrofuran (v/v = 1/3) was employed to extract the pigments, and 30 min of extraction gave satisfactory results with the cells of the day 5 culture. After autoclaving at 134 °C and glucoamylase double digestions, the algal cells with or without the additional corn starch (the internal controls) were smashed and centrifuged to collect clear supernatant for the glucose assay. As shown in Table 2, the measurement accuracies of the additional corn starch (the internal controls) were close to 100% in the six tests using this procedure. The results suggest this procedure can achieve a high level of accuracy for the measurement of endogenous starch in green microalgal cells.

## Discussion

This simple and accurate procedure that was built based on the corn starch internal controls for microalgal starch quantification is also low-cost, due to the use of the thermo-tolerant glucoamylase. This enzyme is able to hydrolyze α-1,6-glucosidic bonds in starch in addition to α-1,4-glucosidic bonds (see the comments of the International Union of Biochemistry of Molecular Biology, IUBMB about this group of enzymes in BRENDA database, and the product information of this enzyme issued by Tokyo Chemical Industry Company). The cost of the enzyme per assay (4 units/assay) in this study was less than USD 0.5 cent, a dramatic difference compared to USD 14.2 using the Sigma-Aldrich starch assay kit and Euro 2.6 using the Megazyme kit purchased in Taiwan. The mass of the internal controls was measured using a high accuracy analytical balance, which gave the readings very close to their true values. This provides the possibility of gauging the accuracy of the results obtained using this quantification method.

In the study of Rose et al. (1991), the “accuracy” of the six starch quantification methods was compared, and up to 40% in variation was found in the results obtained by using those methods. As aforementioned, steps used in those six methods are still adopted recently (Branyikova et al. 2011; Dragone et al. 2011). The accuracy of starch quantifications nowadays is still a great concern. The six methods did not include internal controls or standards of purified starch in those assays. Therefore, the comparisons were actually about the precisions and the result variations measured using those methods. The true values of the starch in their samples were not clear. Therefore, it is unlikely to determine which method was more accurate than the others among those six methods. In the two commercial starch assay kits produced by Sigma-Aldrich and Megazyme companies, the principles the two kits adopt are the same, which are (1) starch degradation to glucose using amylase/amyloglucosidase; (2) glucose conversion to glucose-6-phosphosphate using hexokinase; (3) glucose-6-phosphate conversion to 6-phosphogluconate using glucose-6-phosphosphate dehydrogenase; and (4) measurement of the resulted NADH levels using OD_340_. The two assays do include starch standards, but not internal controls, to calibrate the results from measuring unknown samples. However, the interferences from water contents and other non-glucose components in the starch standards were ignored in the protocols of the two kits. Besides, the three enzyme reactions are also a concern. Generally, more enzyme reactions give more bias in the quantifications.

This quantification procedure has advanced starch assay one step further by using the internal controls compared to other methods. In addition, this method has a high sensitivity. The sensitivity of the DNS method described in this procedure is 28 μg of glucose per reaction at the low end of the standard curve (Fig. 1). One microliter of green microalgal culture in this study had starch that could generate much more than this quantity of glucose. Therefore, this procedure is well suited to the lab scale of microalgal cultivation and starch measurement.

Found in this study, the action of quick-freezing of the cells at − 15 °C right after harvest had positive influence on the accuracy of starch quantification. This might be due to the starch degradation by the endogenous microalgal enzymes at room temperature when the cells were agitated. The measurement accuracy of the internal controls being a little over 100% on average in Table 2 was most likely due to the remaining pigments in the samples. Therefore, double solvent extractions might be necessary if the cell pellets of interest are still green after the first pigment extraction. Complete disintegration of starch granules and thorough glucose polymer digestion are crucial to achieve accurate measurement of starch. High temperature autoclaving at 134 °C and glucoamylase double digestions at 50 °C for more than 20 h yielded satisfactory results. High-speed centrifugation (16,000*g* at room temperature for 10 min) to precipitate the cell debris after the double digestions of the microalgal samples was required to obtain high accuracy of starch measurement.

Although it takes 2 days to quantify microalgal starch using this procedure, the work is not intensive since most time in the 2 days is used for autoclaving (about 4 h from the beginning to the end) and enzyme digestion (one night plus 7 h in the second day). This procedure will be a great tool for the studies of biochemical and genetic engineering that involve in starch biosynthesis or degradation in green microalgae.

## Data Availability

All data generated or analyzed during the current study are included in this published article.
